# 962 This Hair is on Fire! (But it Shouldn’t Be)

**DOI:** 10.1093/jbcr/iraf019.493

**Published:** 2025-04-01

**Authors:** Edgar Villalobos, Casey Kohler, Marcie Lambrix, Monica Gerrek

**Affiliations:** Burn Care Center at MetroHealth; MetroHealth; MetroHealth System Case Western Reserve University; MetroHealth System Case Western Reserve University

## Abstract

Discussion

Published reports of burns resulting from hair styling are increasing but have focused mostly on scald burns that are a result of braiding, whether with natural or synthetic hair. There are a few reports of alternative hair catching fire, however there have been no published reports of the type of scalp burn we have seen multiple times in our Midwest ABA verified burn unit. In one case, a wig caught fire while the patient was lighting a cigarette. In the other cases, the patients’ alternative hair caught fire while cooking. In all cases, the patients had a “band” burn that went from temple to temple across just above their forehead. Due to this unique presentation, we did a literature review to determine the cause and prevalence of these types of burns. While we found no other case studies of this type of burn, we did find that while the alternative hair is well-known to be flammable, the adhesive used to secure it may also be flammable. Notably, there are no flammability standards for adult alternative hair or fixation products, although there are flammability standards for toy products for children 12 and under that include headwear and wigs. Precedent for the lack of standards appears to have been set in 1975 when a designer of male hairpieces was told that the flammable fabrics act did not cover alternative hair, and testing of human or alternative hair products was not required.

According to one source, the alternative hair market size was valued at USD 7.7 Billion in 2022 and is expected to reach a market size of USD 15.7 Billion by 2032. The same report indicates that nearly half of all Americans utilize alternative hair. Other studies show that women, and primarily women of African descent, are driving the demand for these products, especially extensions, although there is an increasing demand by men of African descent. Illness, hair loss and baldness, fashion trends, social media, and use in movies and theatre are other driving forces behind the increase in this market.

Our concern is that consumers are uninformed about the potential hazards of alternative hair. Providers may also be unaware of these issues. For example, most people who use alternative hair use synthetic hair which is less expensive, but more flammable than human hair. In addition, consumers and providers may not realize that the adhesive they use to secure their alternative hair may be flammable and that there are non-flammable options, and they may believe that there are already safety measures in place for alternative hair.

We argue that burn providers and the ABA have an ethical obligation to educate people about the hazards of using alternative hair and to advocate for flammability standards for adult alternative hair and related products. Such advocacy is consistent with other ABA advocacy efforts and with its mission goals of quality care, prevention, and addressing disparities.

